# Axillary lymphoma masquerading as inflammatory breast cancer

**DOI:** 10.2349/biij.2.3.e36

**Published:** 2006-07-01

**Authors:** KL Taubman, MJ McKay

**Affiliations:** 1 Department of Nuclear Medicine, St Vincent’s Hospital, Melbourne, Australia; 2 Divisions of Radiation Oncology and Research, Peter MacCallum Cancer Centre, Melbourne, Australia

**Keywords:** Inflammatory breast carcinoma, lymphoma, chemotherapy

## Abstract

Primary non-Hodgkins lymphoma (NHL) of the breast, and its extranodal spread to the breast resulting from systemic lymphoma, are recognised albeit uncommon conditions. However, lymphoma involving the axilla, presenting with the clinical appearance of inflammatory breast carcinoma (IBC) without infiltration of breast dermal lymphatics has not been reported previously.

As highlighted by the two cases presented here, this entity should be considered in the differential diagnosis of patients presenting with clinical IBC. The cases highlight the importance of careful histological analysis to distinguish IBC from NHL, since management strategies and prognosis are quite different.

## INTRODUCTION

Primary non-Hodgkins lymphoma (NHL) of the breast, and its extranodal spread to the breast as a result of systemic lymphoma, are recognised albeit uncommon conditions [1]. Primary breast lymphoma (PBL) comprises 0.04-0.5% of primary malignant cancers of the breast and 2% of all primary extranodal lymphomas [2].

PBL should be distinguished from the cases presented here, since axillary lymphoma presenting with the clinical appearance of inflammatory breast carcinoma (IBC) has not been reported previously. As highlighted by these cases, this entity should be considered in differential diagnosis of patients afflicted with clinical IBC. The cases highlight the importance of careful histological confirmation in individuals presenting clinically as IBC, since management strategies and prognosis are quite different.

## CASE REPORTS

*Case 1*, a female of 80 years, presented with productive cough, dyspnoea, ankle oedema and weight loss. Her medical history was otherwise largely unremarkable, and her performance status was poor ECOG (Eastern Co-operative Oncology Group – III).

The patient had the classical clinical features of a left-sided IBC, including redness, heat and oedema involving most of the skin of the breast, but without a discreet accompanying breast mass. Mammography and ultrasound of the breast did not detect a mass but the only abnormal feature was thickening of the skin over the involved breast, consistent with dermal oedema. There was an ipsilateral 2.5cm x 2.5cm x 2cm firm, mobile, non-tender left ipsilateral axillary lymph node mass. Chest radiology showed bilateral pleural effusions.

The initial clinical diagnosis was that of IBC with metastatic involvement of the pleural space. Diagnostic and therapeutic pleural aspirations showed atypical lymphocytes but no malignant cells. Multiple large-bore needle biopsies of the skin of the left breast revealed dermal lymphatic ectasia with mild histiocytic perivascular infiltration, but no evidence of malignancy. The size of her axillary nodal mass increased to 7 x 5 cm over a four-day period and then spontaneously decreased to 2 x 1 cm. Interestingly, the breast oedema decreased in severity in parallel to the reduction in size of the nodal mass. Excision biopsy of the axillary lymphadenopathy showed a diffused lymphocytic, well-differentiated (B-cell) non-Hodgkin’s lymphoma (Rappaport Classification); B-cell small lymphocytic lymphoma (WHO Classification).

Investigations at this point, including gallium and CT scanning, and bone marrow analysis, were negative for involvement by lymphoma. The patient was commenced on chlorambucil 10 mg daily and prednisone 60 mg daily for five days, in three-weekly cycles. After one cycle of chemotherapy the patient still had residual small bilateral pleural effusions, but her dyspnoea had improved considerably and her axillary adenopathy had completely resolved, as had the inflammatory signs in her breast.

*Case 2*, a 74 year-old female, presented with pain in the left axilla and, like the first case, clinical inflammatory carcinoma of the left breast, without a palpable mass. As for Case 1, the differential diagnosis included cellulitis of the breast (see Discussion). Firm, irregular, non-tender lymph nodes were palpable in the left axilla (3 x 3 x 2 cm) and in the ipsilateral supraclavicular fossa (2 x 2 x 2 cm). The provisional clinical diagnosis, as in the previous patient, was IBC. Bilateral mammography and ultrasound were negative apart from apparent skin thickening. External examination was otherwise unremarkable. Biopsy of clinically-involved skin over the left breast showed a mild perivascular lymphocytic infiltrate, without definite tumour cells. A left supraclavicular fossa lymph node excision biopsy was performed; whose pathological examination showed follicular mixed NHL.

Staging investigations at this point were negative for systemic lymphoma involvement. She was commenced on intravenous and oral chemotherapy comprising CVPP (cyclophosphamide 1,000 mg day 1, vincristine 2 mg day 1, procarbazine 150 mg orally and prednisone 60 mg per day orally for seven days each). She received four one-monthly cycles and achieved a complete clinical remission after one cycle.

## DISCUSSION

To our knowledge, these are the only two cases of lymphoma involving the axilla which have clinically simulated IBC at presentation. The cases demonstrate the importance of careful histopathology in distinguishing IBC with or without axillary lymphadenopathy from lymphoma involving the axilla.

**Figure 1:**
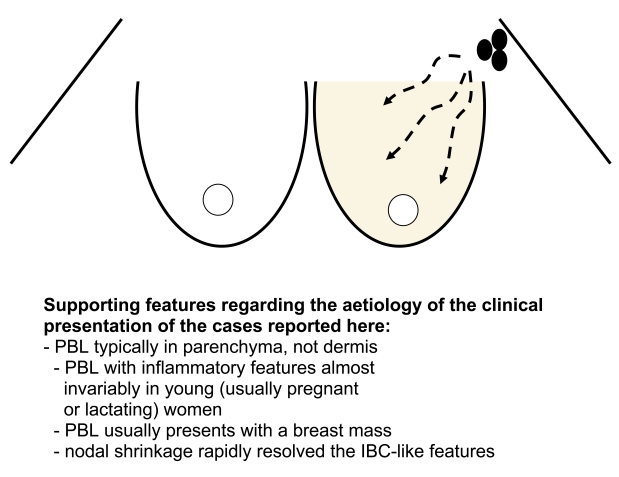
An hypothesis for the aetiology of clinical features of inflammatory breast cancer in the cases reported here.

It should be noted that it is only for the last decade or so that such clinical features of IBC have required a corroborating histopathological diagnosis. In the cases presented here, obtaining histology of the masses in the regional lymph node basins was essential to achieving correct diagnosis, as multiple cutaneous biopsies were negative for malignancy. The present cases should also be distinguished from primary breast lymphoma (PBL; Table 1 and Figure 1).

**Table 1:** A comparison of the clinicopathological features of inflammatory breast cancer (IBC), primary breast lymphoma (PBL) and axilliary lymphoma presenting as IBC (AL). IBC data was as reviewed by Giordano and Hortobagyi [3]; PBL data was from Gholam, et al. [1] and Maounis, et al. [2]; AL data was from the two cases presented here. U/K: unknown; +: typically present; -: absent.

	**IBC**	**PBL**	**AL**
**Age**	Spectrum: from younger to older	Two peaks: younger or older	Older
**Palpable or mammographically-visible mass**	+/-	+	-
**Bilaterality**	Rare	Not uncommon	U/K
**Clinical lymph node involvement**	+/-	+	+
**Typical histology**	Invasive ductal adenocarcinoma	Younger: Burkitt or Burkitt-type (B-cell); Older: NHL, diffuse large (B-cell)	B-cell small lymphocytic or follicular mixed NHL
**Rapid treatment response**	Uncommon	+	+

The presentation of both individuals fitted the clinical criteria for a diagnosis of IBC, namely, signs of inflammation (redness, heat, induration) skin oedema /*peau d’orange* with or without tenderness, and a cutaneous erysipeloid edge. To fit the contemporary definition of IBC, these changes must be present in greater than one-third of the breast, as they were in these cases; however, a palpable mass need not necessarily be present [3].

The differential diagnosis for these cases included cellulitis of the breast. Common features of breast cellulitis include a sudden onset, usually accompanied by constitutional symptoms such as fever and chills and mild cutaneous oedema; leukocytosis is usually present. The cases here had breast oedema but none of the other signs of breast cellulitis.

Breast cellulitis typically begins in the pre-menopausal setting and particularly during lactation [4]. In contrast, the cases presented here also lacked such features. Additionally, the size of axilliary adenopathy in both patients, as well as the pleural effusions in Case 1, was more consistent with a malignant aetiology. Nevertheless, the differential diagnosis of breast cellulitis should be entertained at least until histo- or cyto- logical diagnosis confirms the presence of malignant cells.

Histologically, IBC is characterised by lymphatic infiltration by adenocarcinoma cells, with plugging of dermal lymphatics by tumour emboli. The latter are largely responsible for the clinical manifestations of IBC, secondary to lymphatic backpressure.

In IBC, the disease spreads predominantly via lymphatics, and oedematous skin, and if biopsied, typically shows adenocarcinoma cells in the lymphatics [3]. It is well known that oedema of the arm and/or breast can occur after axillary dissection and/or radiotherapy to the axilla, or in advanced IDC, due to lymphatic disruption [5].

Involvement of the axillary nodes by lymphoma could presumably also interfere with lymphatic function. In the two cases of axilliary lymphoma (AL) simulating IBC, multiple biopsies of areas of breast skin affected by *peau d’orange* failed to reveal malignant cells but simply showed an inflammatory cell infiltrate. We postulate that the oedematous, inflamed breasts in our patients were due to lymphatic obstruction by lymphoma in the axilla, with retrograde pressure build-up in lymphatics causing the physical signs in the breast. This finding would be atypical for IBC. Some other features of the two cases were different to IBC: one individual showed spontaneous regression of the axilliary nodal mass (i.e. prior to therapy), and lymph node masses were firm rather than hard (the latter expected in carcinomas) and both individuals had dramatic early responses to relatively simple chemotherapy regimens.
